# Resilience to depression: the role of benevolent childhood experiences in a South African sample

**DOI:** 10.3389/fpsyg.2023.1209504

**Published:** 2023-07-21

**Authors:** Oluwaseyi Dolapo Somefun, Linda Theron, Jan Höltge, Michael Ungar

**Affiliations:** ^1^Department of Educational Psychology, University of Pretoria, Pretoria, South Africa; ^2^Department of Psychology, University of Hawai’i at Mānoa, Honolulu, HI, United States; ^3^Faculty of Health, Dalhousie University, Halifax, NS, Canada

**Keywords:** young adults, benevolent childhood experiences, resilience, South Africa, adversity

## Abstract

**Background:**

Studies elsewhere show that benevolent childhood experiences (BCEs) have protective mental health value. However, this protective value has never been investigated in an African context. Given the need to better understand what might support mental health resilience among African young people, this study explores the relationship between BCEs and depressive symptoms among a South African sample of young adults living in a community dependent on the economically volatile oil and gas industry.

**Methods:**

A sample of young adults in an oil and gas community in South Africa (*N* = 313, mean age 20.3 years, SD = 1.83, range from 18 to 26; majority Black African) completed self-report questionnaires to assess BCEs and depressive symptoms (Beck Depression Inventory-II). The analysis controlled for socio-demographics and experience of family adversity. Multinomial logistic regressions were used to examine the association of BCEs with depressive symptoms using STATA 17.

**Results:**

The majority (86.4% of the sample) reported all 10 BCEs. Of the 10 BCEs, having at least one good friend was the most reported (94%) compared to 75% of the sample reporting having a predictable home routine, such as regular meals and a regular bedtime. The unadjusted multinomial logistic regression analysis indicated that having at least one good friend, comforting beliefs, and being comfortable with self were associated with lower odds of moderate depression. The adjusted results showed no association between BCEs and the depression of young adults in this sample.

**Conclusion:**

In this South African sample, our results do not show protective associations between BCEs and depression. This could be as a result of the homogeneity in our sample. It is also possible that the BCEs explored could not counteract the effect of chronic risk factors in the lives of the young people in this study context. Further research is needed to understand this complexity.

## Introduction

Benevolent early life experiences, known as BCEs, are enabling experiences before the age of 18 that foster positive perceptions of safety, security, connectedness, and predictability during development (Narayan et al., 2018). BCEs, such as access to supportive adults or having good neighbors, have been found to have a positive impact on the development and well-being of adults who were exposed to hardship during their childhood (Narayan et al., 2018; Crandall et al., 2019; Daines et al., 2021). For this reason, BCEs are associated with resilience (i.e., the capacity for positive outcomes – such as mental health – despite exposure to significant risk; Masten, 2014). However, little is known about the potential long-term protective effects of BCEs on the mental health outcomes of young adults in South Africa. This paper redresses this existing research gap among a sample of emerging adults aged 18–26 years old.

The mental health outcomes of adolescents and youth globally are an important cause for concern, with mental health disorders contributing significantly to the global burden of disease (Wies et al., 2021). Depression, which is a leading contributor to this burden, has shown alarming upward trends among youth (Thapar et al., 2022). While only a limited number of studies have investigated mental illness among young people in sub-Saharan Africa (Steel et al., 2022), a narrative review of 37 of these studies showed a median point depression prevalence of 26.9% for sub-Saharan young people aged 10–19 in general and 29% for those with experiences of significant risk exposure (e.g., violence or poverty) (Jörns-Presentati et al., 2021). This incidence is comparable to that reported in global prevalence studies (Shorey et al., 2022). Protecting the health and well-being of young adults is important, not least because this has been linked with socio-economic development (Nyundo et al., 2020). This is because of the association between the mental health of youth cohorts and other outcomes such as their self-esteem, interaction with their family members and peers, and gainful employment.

Likewise, there is concern about the mental health of South African young people. The available nationally representative data on mental health in South Africa comes from the National Income Dynamics Study (NIDS). The 2014–2015 data reveals that 26% of the population have significant depressive symptoms (Mungai and Bayat, 2019), which is similar to the prevalence among youth aged 15–24 (Somefun and Simo Fotso, 2020). Despite the high prevalence of depression among young people in South Africa, significant barriers exist in accessing mental health services (Craig et al., 2022). Stigma surrounding mental health issues is widespread, particularly in rural areas, and many young people may be reluctant to seek help due to fear of discrimination or judgment (Guttikonda et al., 2019). The shortage of mental health professionals and limited resources also make it challenging for young people to access adequate treatment and support in South Africa (Mokitimi et al., 2019). Studies have posited that prevention of mental health disorders is important because of the effect they have in later adulthood (Sorsdahl et al., 2021).

The causes of depression among youth in South Africa are diverse and complex. Socioeconomic factors such as poverty, unemployment, and social inequality are significant contributors to depression among young people (Hatcher et al., 2019; Somefun and Simo Fotso, 2020). Many young people in South Africa experience high levels of stress and anxiety due to the challenges of daily life, including violence, crime, and social isolation (Scorgie et al., 2017; Sui et al., 2021). In addition to the external factors contributing to depression, there are also internal factors that can lead to depression among young people. Genetic predisposition (Alshaya, 2022), past traumatic experiences, and chronic stress (Thapar et al., 2022) can all increase the risk of developing depression. The impact of the COVID-19 pandemic has also contributed to the increase in depression among young people in South Africa (Haag et al., 2022). The pandemic has resulted in increased social isolation, economic uncertainty, and disrupted education, which has taken a toll on the mental health of young people (Theron L. et al., 2021).

### The protective role of benevolent childhood experiences

Missing in the literature about the depression of emerging adults in South Africa is the effect of BCEs on the mental health of young adults in South Africa. In particular, it is unclear whether experiences of benevolence during childhood can protect against the development of depression in young adulthood in South Africa. As noted earlier, BCEs are favorable childhood experiences (i.e., from birth to the age of 18), including but not limited to a stable and supportive family environment, access to quality education, positive peer relationships, opportunities for creative expression and play, healthy nutrition, and safe living conditions (Narayan et al., 2018, 2023). BCEs are critical to a child’s overall development and well-being, providing a foundation for future success and resilience. Aligned with social-ecological and multisystemic approaches to resilience (Ungar and Theron, 2020; Masten et al., 2021), BCEs shift the focus away from the individual child and their inherent strengths to resources in the child’s everyday life that enable wellbeing. Unlike family adversity, BCEs are usually not associated with the socioeconomic status of the individual (Hou et al., 2022).

Theoretically, Narayan et al. (2021) argue that childhood experiences exert a formative and enduring influence on adaptation and maladaptation throughout the lifespan and across generations. Other theories confirm that benevolent childhood experiences serve as protective factors against depression and anxiety. The multisystemic resilience framework (Zimmerman, 2013; Masten and Cicchetti, 2016; Ungar and Theron, 2020; Ungar, 2021), which frames the study we report, and the positive youth development framework (Lerner et al., 2009) adopt multisystem-informed and strength-based approaches. These frameworks highlight the strengths within children and systems in their surrounding environments, including family, school, and neighborhood, as crucial elements in safeguarding them from risks and facilitating their adaptation in the face of adversity.

For instance, in a study focused on family system factors and conducted in the United States, it was found that strong parental support and minimal parent–child conflict, reflecting parental approval, were significantly associated with enhanced mental health outcomes among adolescents (Chen and Harris, 2019). Similarly, schools represent an important environment where children spend a significant amount of their time during late childhood. A Study using Census data from the National Longitudinal Study of Adolescent Health in the United States showed a correlation between negative school experiences, particularly peer rejection and bullying, and the development of depression and anxiety among adolescents (Coley et al., 2018). Another United States-based study found that a strong sense of school connectedness reduces the likelihood of young people experiencing adolescent depression and anxiety (Shochet et al., 2006). At the community level, experiencing victimization has been linked to a higher likelihood of depression among young adults in South Africa (Somefun et al., 2023). This finding contrasts with a study conducted among adolescents, which found that a high level of neighborhood efficacy, measured as trust in the neighborhood, did not directly impact depressive symptoms in adolescents (Choi et al., 2021).

Other studies show that BCEs have a profound impact on an individual’s mental and emotional well-being in adulthood. For example, a study by Narayan et al. (2019) explored the benefits of BCEs and fathers’ perspectives among pregnant women in the United States. The study aimed to identify BCEs and other positive experiences that these families had, in order to develop interventions that build on their existing strengths and resources. The authors concluded that therapeutic interventions that leverage BCEs and involve fathers can promote resilience and positive outcomes for low-income families. Another study (Merrick et al., 2019) working with an ethnically diverse homeless adult population in the United States found that BCEs were associated with several positive outcomes, including lower levels of psychological distress and parenting stress, and higher levels of perceived social support. Similar results were found among a sample of university students in Western USA during the early stages of the pandemic (Doom et al., 2021). Using an online survey, this study found that BCEs were associated with wellbeing during the pandemic, including lower levels of depression and loneliness. Using an online survey to collect data from a large sample of US adults (18–58 years) during the early months of the pandemic, (Doom et al., 2021) highlighted that the COVID-19 pandemic may have exacerbated the impact of childhood experiences on mental health outcomes, and that both adverse and positive childhood experiences may be associated with stress and uncertainty. They also suggest that interventions aimed at promoting BCEs may be an effective way to support resilience and well-being during and after the pandemic. This implies that individuals who report more BCEs are less likely to experience mental health issues, such as anxiety and depression, and more likely to have higher levels of well-being.

A study with 275 adults living in Scotland reported similar positive associations between BCEs and wellbeing (Karatzias et al., 2020). Specifically, BCEs were associated with fewer disturbances in self-organization (e.g., a negative self-concept or emotional dysregulation). BCEs were similarly protective of psychological wellbeing in a study with 1816 undergraduate university students in China (Hou et al., 2022). Another Chinese study, this time with a large sample of students (average age: 20) from the cities of Xuzhou, Nanjing and Wuhan, reported an association between higher levels of BCEs and lower levels of depression and suicidal ideation. Overall, international studies confirm the protective effects of BCEs on young adult/adult mental health and call for greater facilitation of BCEs.

In summary, BCEs are believed to shape the development of essential psychological and emotional skills during childhood. They support the development of positive factors such as healthy attachment, emotional regulation, and interpersonal relationships that provide a solid foundation for adaptive coping mechanisms and resilience. These foundational skills are crucial in navigating challenges and reducing the risk of developing depressive symptoms later in life. They also act as protective factors against the onset and progression of depression. Supportive and nurturing environments during childhood foster a sense of security, belonging, and self-worth, which in turn can buffer individuals against stress, adversity, and negative life events. By promoting psychological well-being and fostering a positive self-image and sense of belonging, BCEs can reduce the vulnerability to depressive symptoms.

### The present study

As demonstrated, although there is international empirical evidence that BCEs protect mental health, no such empirical study has yet been conducted in sub-Saharan Africa, including South Africa. Understanding the potential protective role of BCEs on depression among young adults in South Africa is crucial given the high prevalence of depression and scarcity of professional mental health support in this country. It is also important because promoting BCEs can be a cost-effective way to prevent mental health challenges from developing in the first place. Investing in interventions and programs that support BCEs can help promote mental health and prevent mental health challenges. By preventing mental health challenges from developing in the first place, we can reduce the need for more expensive interventions, such as mental health treatments and hospitalizations.

The aim of this article is to examine the apparently protective BCE-depression relationship, using a sample of young adults from a structurally violent community in South Africa. Young people in stressed South African communities are potentially more vulnerable to depression and have less access to mental health services than those in more privileged communities (Mindu et al., 2023). To achieve this aim, we examine the combined effect of BCEs on depression and also examine the influence of single BCEs on depression. We anticipated that higher levels of total BCEs would have significant protective effects given the understanding that more resources (e.g., more BCEs) are typically associated with better youth outcomes (Merrick et al., 2019; Doom et al., 2021; Hou et al., 2022). Given the prominence of relational resources – especially nurturing or mentoring care from an adult – to the resilience of South African young people (Van Breda and Theron, 2018), we hypothesized that two specific BCEs (i.e., being safe with a caregiver and having a supporting adult or caregiver) will show significant protective associations with the depression outcomes of young adults in our sample. In summary, we aim to answer the following questions:

What is the relationship between protective factors (BCEs) and depression in young adults from a structurally violent community in South Africa?
Do higher levels of total BCEs have a significant protective effect on depression?
Do specific BCEs, such as feeling safe with a caregiver and having a supporting adult, show significant protective associations with depression outcomes in young adults?
By doing so, this article seeks to contribute to the growing body of research on the importance of early life experiences for mental health outcomes in emerging adulthood in South Africa.

## Method

### Procedure

We used data from the Resilient Youth in Stressed Environments (RYSE) study. The RYSE study was a 5-year (2017–2022) study that investigated the resources that support youth resilience in stressed communities in Canada and South Africa (Ungar et al., 2021). Ethical approval was obtained from the Institutional Review Boards of the universities where the principal investigators are affiliated in Canada (Health Sciences Research Ethics Board, Dalhousie University, #2017-4321) and South Africa (Faculty of Health Sciences Research Ethics Committee, University of Pretoria, #UP17/05/01).

This paper focuses on the data collected in South Africa because of the paucity of research examining benevolent childhood experiences of young people in African contexts. The target communities, Secunda and eMbalenhle, for this study were communities that were chiefly dependent on the local oil and gas industry and were vulnerable to the risk associated with boom-and-bust economic cycles (Ungar et al., 2021). At the time of the study, young adults in the study sites faced a myriad of challenges such as high rates of unemployment, structural violence, family conflicts, exposure to poverty and lack of access to basic services and related violent protests. As in many resource-constrained communities in South Africa (Canham, 2018), these challenges are chronic. Their effects are also potentially worse for emerging adults seeking to fulfill developmental milestones, such as finding stable employment or starting a family, with many emerging adults reporting related psychological distress (Theron L. C. et al., 2021; Theron and Ungar, 2022).

Participants meeting specific eligibility criteria were intentionally recruited by study gatekeepers. These criteria included being between the ages of 14 and 24 at baseline, residing in either of the study communities, having direct or indirect experience (positive or negative) of petrochemical industry impacts (such as personal or family layoffs related to the industry or involvement in industry-sponsored community investment programs), and possessing English literacy skills. To reach a wider audience, the study team advertised the research, including the inclusion criteria, in local schools and popular shops. Furthermore, participants were given the opportunity to nominate eligible peers for potential inclusion in the study.

Trained research assistants administered the survey to participants who had consented to be part of the RYSE study. Mostly, this was accomplished through face-to-face interactions using interview-style methods for data collection.

### Participants

The 2018 survey sample included a total of 600 young people aged 14–24. For the purposes of this paper, we focused on the young adults (18–26-year-olds) in this sample (*n* = 313) since the aim was to investigate the association between BCEs (experienced before the age of 18) and depression. The mean age of the current sample was 20.31 (SD = 1.83). There was a similar distribution of females (*n* = 159, 51.3%) and males (*n* = 150, 48.4%). Most participants self-identified as being Black/African (*n* = 307, 98.4%).

### Measures

#### Depression

We used the Beck Depression Inventory-II (Beck et al., 1996) to measure depression symptoms in the two-week period before the survey. The BDI-II is a widely used 21-item self-report inventory measuring the severity of depression in adolescents and adults. This instrument has been validated among University students in South Africa (Makhubela and Mashegoane, 2016). It has 21 items specific to a symptom of a depression with a 4-point (0–3) scale to examine severity. The reliability coefficient was satisfactory, *α* = 87. Items were summed up and categorized as per the inventory’s manual: 0–13 “Minimal depression,” 14–19 “Mild depression,” 20–28 “Moderate depression” and 29–65 “Severe depression.”

#### Benevolent childhood experiences

To measure BCEs, we used the (Narayan et al., 2018) original BCEs scale (Narayan et al., 2018). This scale is a checklist of 10 positive childhood experiences occurring between birth and 18 years. The items in this scale include (1) having at least one safe caregiver, (2) having at least one good friend, (3) having beliefs that gave comfort, (4) enjoying school, (5) having at least one teacher who cared, (6) having good neighbors, (7) having an adult (not a parent/caregiver) who could provide support or advice, (8) having opportunities to have a good time, (9) having a positive self-concept, and (10) having a predictable home routine. Answers to these questions were dichotomous (Yes or No). Items were summed with higher scores indicating higher experience of BCEs.

#### Family adversity

We adapted the Life events questionnaire by Labella et al. (2019). Participants were asked 10 questions measuring different types of adversities. These questions measured experience of adverse events in the family such as death, divorce/separation of parents, violence, parental mental and physical illness, foster parenting, and parental incarceration. Responses were binary (yes and no) and the sum score range from 0 to 10. Items were summed and higher scores indicated high levels of family adversity.

#### Covariates

Age (continuous) and gender (female vs. male) were included in the analysis.

### Statistical analysis

Descriptive analyses were used to examine the frequency distribution of the variables used in the study. We then used multinomial logistic regression to determine the unadjusted association between BCEs and depression. Unadjusted regression is used when there are no confounding variables or when the goal is to examine the relationship between two variables without controlling for other potential factors. The adjusted regression, on the other hand is used when there are potential confounding variables that might affect the relationship between the independent and dependent variables. By adjusting for these confounding variables, the regression model can better isolate the true relationship between the independent and dependent variables.

This was done in two models. In the first model, we examined the association between a composite measure of BCEs (i.e., a total BCE score) and depression and in the second model, we examined the association between each individual BCE item and depression. The minimal depression sub-group served as the reference category in each model. Multinomial logistic regression is an extension of binary logistic regression that allows for more than two categories of the dependent or outcome variable. This model also uses maximum likelihood estimation to evaluate the probability of categorical membership, like binary logistic regression. It does not assume normality, linearity, or homoscedasticity (Starkweather and Moske, 2019). All analyses were conducted using STATA statistical software version 17.

## Results

As summarized in Table 1, most young adults reported minimal to mild symptoms of depression. Most reported positive childhood experiences as well as high levels of family adversity. 86.4% of the sample reported all 10 BCEs.

**Table 1:** Descriptive statistics among study variables.

Variable	Frequency (%)	Mean (SD)
Depression (Full sample)		13.20 (8.64)
Minimal	168 (56.38)	7.07 (3.50)
Mild	67 (22.48)	15.93 (1.64)
Moderate	43 (14.43)	23.60 (2.41)
Severe	20 (6.71)	33.35 (2.87)
Felt safe with caregiver		
No	13 (4.17)	
Yes	299 (95.83)	
Had at least one good friend		
No	20 (6.41)	
Yes	292 (93.59)	
Comforting beliefs		
No	32 (10.26)	
Yes	280 (89.74)	
Liked school		
No	72 (23.08)	
Yes	240 (76.92)	
Teacher who cares about you		
No	43 (13.78)	
Yes	269 (86.22)	
Good neighbors		
No	67 (21.47)	
Yes	245 (78.53)	
Had adult (not parent/caregiver) who could give support or advice		
No	30 (9.62)	
Yes	282 (90.38)	
Opportunities to have a good time		
No	39 (12.58)	
Yes	271 (87.42)	
Liked or felt comfortable with self		
No	12 (3.85)	
Yes	300 (96.15)	
Had a regular routine		
No	78 (25.00)	
Yes	234 (75.00)	
Family adversity		
Low	83 (27.21)	
High	222 (72.79)	
Gender		
Female	159 (51.46)	
Male	150 (48.54)	
Age		20.31 (1.83)

Model 1 investigated the unadjusted association between the composite measure of BCEs and depression. As shown in Table 2, there was no significant association between the combined BCE scores and depression. Participants with high BCEs had lower odds of being moderately depressed compared to their counterparts with low BCEs, but this association was not statistically significant.

**Table 2:** Unadjusted association between BCE total score and depression (Model 1 unadjusted).

Depression Minimal depression (RC)	RRR	95% CI
Mild depression		
BCE	0.82	0.43–1.53
Family adversity	1.27	0.66–2.43
Gender	0.80	0.44–1.45
Age	0.91	0.77–1.08
Moderate depression		
BCE	0.41**	0.17–0.97
Family adversity	1.95	0.83–4.60
Gender	0.40	0.19–0.84
Age	1.02	0.84–1.25
Severe depression		
BCE	0.90	0.30–2.64
Family adversity	1.16	0.37–3.58
Gender	0.09***	0.02–0.42
Age	1.09	0.82–1.45

**p < 0.05; ***p < 0.01. Blank items are reference categories.

Model 2 (see Table 3) investigated the unadjusted association between individual BCE item scores and depression. As shown in Table 3, three specific items (i.e., having at least one good friend, comforting beliefs, and being comfortable with themselves) were significantly associated with lower odds of moderate depression.

**Table 3:** Unadjusted association between individual BCE item scores and depression (Model 2 unadjusted).

Depression Minimal depression (RC)	RRR	95% CI
Moderate depression		
Had at least one good friend	0.25***	0.08–0.75
Comforting beliefs	0.33**	0.12–0.89
Liked or felt comfortable with self	0.17**	0.03–0.82

**p < 0.05; ***p < 0.01. Blank items are reference categories.

Table 4 shows the adjusted association between the combined BCE scores and depression. There was no significant association between combined BCE scores and depression. Gender was significantly associated with moderate and severe depression: young males had lower odds of depression compared to their female counterparts.

**Table 4:** Adjusted association between BCE total score and depression (Model 1 adjusted).

Depression Minimal depression (RC)	RRR	95% CI
Mild depression		
BCE	0.75	0.40–1.38
Family adversity	1.25	0.65–2.38
Gender	0.79	0.45–1.40
Age	0.90	0.77–1.06
Moderate depression		
BCE	0.40	0.17–0.97
Family adversity	1.82	0.78–4.23
Gender	0.45*	0.22–0.91
Age	0.96	0.80–1.16
Severe depression		
BCE	0.81	0.29–2.25
Family adversity	1.24	0.42–3.63
Gender	0.09***	0.02–0.40
Age	1.04	0.81–1.33

*p < 0.1; ***p < 0.01. Blank items are reference categories.

For the adjusted association (Table 4), there was no significant association between individual BCE item scores and depression.

## Discussion

This study examined whether reported BCEs were associated with depression levels reported by young adults in the South African RYSE sample. No previous African study has explored the potentially protective effects of BCEs for African young adults. In African contexts, including South Africa, young people in chronically resource-constrained communities are at elevated risk for depression and have little access to mental health supports (Tomita et al., 2017; Haag et al., 2022). This reality compels attention to ordinary resources (like BCEs) that might mitigate this mental health risk.

Studying benevolent childhood experiences (BCEs) and their relationship with depression among young adults in a violent and chronically disadvantaged setting in South Africa is crucial for several reasons. Firstly, by examining the role of BCEs in this setting, we can gain insights into the potential protective factors that may mitigate the negative impact of violence and ongoing disadvantage on adolescent mental health. Exposure to violence and ongoing disadvantage are associated with continuous traumatic stress (Eagle and Kaminer, 2013). Put differently, we can identify which BCEs, if any, have a mental health advantage when young people are exposed to continuous traumatic stress Identifying specific BCEs that are associated with lower rates of depression can inform interventions and programs aimed at promoting resilience and well-being in similar contexts.

In addition, this research contributes to the growing body of literature on the impact of childhood experiences on mental health outcomes, particularly in challenging environments. It provides an opportunity to expand our understanding of the complex interplay between BCEs, violence, and depression among young adults, filling gaps in knowledge and enriching the field of mental health research. This is particularly important when exposure to significant stress is protracted.

Our results are unexpected. There was no support for our expectation that young people reporting higher cumulative levels of BCEs would report better mental health, even though young people reported high levels of BCEs. This finding is surprising given the widespread poverty, violence, and other challenges that many South African children face and the understanding that socioeconomic context can negatively impact access to childhood resources, such as caring parents or predictable family routines (Conger and Donnellan, 2007). However, several factors may explain why these young adults reported benevolent experiences in their childhood.

Firstly, South Africa has a rich cultural tradition that places a strong emphasis on community and family (Ramphele, 2012). This cultural emphasis may have contributed to the positive childhood experiences reported by the emerging adults in this study. For example, close family ties and social support from extended family members may have provided a buffer against the negative effects of poverty and other stressors.

Secondly, the emerging adults in this sample may have been more likely to perceive their childhood experiences positively due to a cognitive bias known as the “positivity effect.” The positivity effect refers to the tendency for individuals to remember and focus on positive events and experiences more than negative ones (Reed and Carstensen, 2012). This bias may have influenced the way that the emerging adults in this study remembered and reported their childhood experiences, leading to an overestimation of benevolent experiences.

Finally, it is possible that the emerging adults in this sample reported high benevolent childhood experiences because of social desirability bias. Social desirability bias refers to the tendency for individuals to present themselves in a positive light in social situations (Latkin et al., 2017). The emerging adults in this study may have felt pressure to report positive childhood experiences in order to conform to social norms and expectations.

Regarding our surprise that BCEs showed no significant protective effects, it is possible that cumulative positive childhood experiences were not sufficient to mitigate the compound risks participants were exposed to and experienced (i.e., a chronically stressed community in combination with high levels of family adversity). Because this exposure continued into early adulthood (a demanding developmental stage that is associated with heightened psychological distress when developmental milestones cannot be met; Arnett, 2000), it possibly negated the protective effects of a healthy childhood. There has been some concern that resilience studies that focus narrowly on single adaptive outcomes (e.g., negligible symptoms of depression) might have misrepresented the notion that resilience is commonplace (Infurna and Luthar, 2018). Perhaps the opposite applies in this case: BCEs might have demonstrated protective effects if we had investigated more than a single adaptive outcome, or at least an outcome that might fit better to the studied context.

We posited that feelings of safety and adult support would have pronounced protective mental health effects for young adults in our sample compared to other BCEs. Our expectation related to the prominence of relational resources – especially nurturing or mentoring care from an adult – to the resilience of South African young people (Van Breda and Theron, 2018), including older adolescents’/young adults’ mental health (Theron et al., 2022). However, the BCEs associated (albeit non-significantly) with lower levels of depression for young adults in our study were: having at least one good friend, comforting beliefs and being comfortable with oneself. Certainly, prior resilience studies in South Africa do acknowledge the enabling value of personal strengths (such as self-acceptance; Van Breda and Theron, 2018), good friends (with emphasis on these friends being trustworthy and prosocial; e.g., Van Breda and Dickens, 2017; Singh and Naicker, 2019) and comforting beliefs (typically faith-based ones; e.g., Mhaka-Mutepfa and Maundeni, 2019). Given this, we were surprised that the association reported was non-significant.

Overall, we wondered whether we might have found significant associations for the cumulative BCEs and/or single BCEs if the scale had included additional positive childhood items that resonate with African culture. The measured BCEs may not have been context specific enough or too broad to have captured important nuances in participants’ experiences. For instance, African children are socialized to value interdependence with people beyond their immediate social connections (family, friends, neighbors and teachers), to appreciate organized religion and spiritual practices (Brittian et al., 2013; Balton et al., 2019), and to engage in cultural rituals (e.g., coming of age celebrations) (Ramphele, 2012). All the aforementioned are potential sources of positive childhood experiences not documented in the Narayan et al. (2018) BCE scale. Similarly, African children in resource-constrained communities might not be familiar with daily routines that include regular mealtimes, but they are likely to be familiar with daily routines that include opportunities to contribute constructively to their household (e.g., chores) and to engage informally in cultural/sporting activities with other children in the neighborhood. It would be valuable to trial BCE items that better fit African children’s context and determine the associations of such contextually relevant items with mental health outcomes.

The multisystemic resilience literature is encouraging attention to resources in the physical environment, too (Ungar and Theron, 2020). This relates to growing understandings that the built environment (e.g., quality housing) and natural environment (e.g., green spaces to play) matter for young people’s positive outcomes (Adams et al., 2017; Pillay, 2017). Children’s positive childhood social experiences are often intertwined with these spaces (e.g., opportunities to play; Adams et al., 2017). It might, therefore, be valuable to adapt the BCE scale so that is both contextually relevant and appreciative of the spaces/places that facilitate positive childhood experiences (e.g., Have you had spaces where you could play safely?).

### Limitations

It is possible that our small sample size was not large enough to reveal any statistical power between the variables. When a sample size is too small, the study may lack sufficient statistical power to detect a significant relationship or difference between the variables, even if there is a true effect (Faber and Fonseca, 2014). In other words, the sample size may not be large enough to provide a reliable estimate of the true population parameters. Another reason for these results could be that young adults experiencing depression may have negative biases in how they recollect issues that took place during their early childhood which may result in remembering more negative experiences compared to positive ones. In addition, majority of our sample reported high BCEs which highlights the homogeneity of the population sampled and may the reason why the results showed no significant differences.

## Conclusion

Previous studies have suggested that BCEs may have a significant positive impact on mental health outcomes (Narayan et al., 2019; Karatzias et al., 2020; Doom et al., 2021; Hou et al., 2022), but our findings did not support this hypothesis. Our study, which was the first to use a sample of youth in South Africa to examine these associations, encourages attention to how context might shape the protective effects of BCEs. While BCEs may play a role in shaping cultural and social identity, they may not have a significant impact on mental health outcomes in the absence of other factors like large-scale redress of structural violence. Further research is needed to better understand the complex interplay of cultural, social, and psychological factors that inform contextually meaningful BCEs and contribute to mental health outcomes among youth, also in Africa.

## Data availability statement

The raw data supporting the conclusions of this article will be made available by the authors, without undue reservation.

## Ethics statement

The studies involving human participants were reviewed and approved by Health Sciences Research Ethics Board, Dalhousie University, #2017-4321 and Faculty of Health Sciences Research Ethics Committee, University of Pretoria, #UP17/05/01. The patients/participants provided their written informed consent to participate in this study.

## Author contributions

OS and LT contributed equally to the conceptualization of the manuscript and the background of the study. OS was responsible for the data analysis and interpretation of results. LT and JH contributed to the interpretation of the results. All authors of the manuscript contributed to the study design, data collection, data analysis, interpretation of results, manuscript preparation, involved in the manuscript preparation, reviewed, and approved the final version of the manuscript for submission.

## Conflict of interest

The authors declare that the research was conducted in the absence of any commercial or financial relationships that could be construed as a potential conflict of interest.

## Publisher’s note

All claims expressed in this article are solely those of the authors and do not necessarily represent those of their affiliated organizations, or those of the publisher, the editors and the reviewers. Any product that may be evaluated in this article, or claim that may be made by its manufacturer, is not guaranteed or endorsed by the publisher.
